# Keeping Up with the Times

**DOI:** 10.5195/cajgh.2012.39

**Published:** 2012-10-22

**Authors:** Valentina Burambayeva

**Affiliations:** Head of the Department of Organizational and Analytical Work; Quality Manager of the Center for Sanitary and Epidemiological Expertise, Mangistau region

**Keywords:** news, implementation of standards

The Republic of Kazakhstan is in transition to the World Trade Organization (WTO) accession. Accessing to WTO has always been and remains a foreign policy priority for Kazakhstan. As a part of this transition, Kazakhstan is identifying new directions for developing and reforming healthcare disciplines. The goal of this overview is to provide information about the work of centers of sanitary and epidemiological expertise to medical and public health professionals in various fields. Additionally, the goal of this paper is to provide information about international accreditation and quality control activities of the centers.

There are 16 centers of sanitary and epidemiological expertise in the Republic, 15 of which have been locally accredited. Implementation of innovation policy at the centers for sanitary and epidemiological expertise fueled the process of international accreditation. It is important to point out that the only center which has an international accreditation as of September of 2012 is the Astana city center.

The center for sanitary and epidemiological expertise of Mangistau region was accredited in 2010 and gained the status as a test center. The Mangistau test center applies previously developed criteria and procedures to estimate technical competence. One of the main standard documents used to estimate competence is ISO/IEC 17025 from the International Organization for Standardization/International Electrotechnical Commission, which assesses the following factors:

- personnel technical competence
- validation of testing methods
- efficiency, calibrating test and service of testing equipment
- testing environment
- selection, processing and transportation of testing samples
- quality provision of testing and calibration data

The introduction of a quality management system was a success. In order to increase the technical competence level, 29 specialists from the center have been trained and have received certificates of compliance.

In September 2011 and April 2012, the Ministry of Healthcare of the Republic of Kazakhstan within the framework of the World Bank project, component F (“Food Safety Program and World Trade Organization accession”), arranged international workshops aimed to train quality managers to meet international requirements and standards. Training seminars for international accreditation provided hands on and methodological sessions combined with the dissemination of the comprehensive tutorial materials. Regional trip seminars were organized by the center specialists.

Laboratories of the Republic were accredited by the National Center for Accreditation (NCA). On October 27, 2010, NCA signed the International Laboratory Accreditation Cooperation Mutual Recognition Arrangement (ILAC MRA) agreement on mutual international acceptance of ILAC standards.

All accredited testing centers for sanitary and epidemiological expertise have an agreement with the National Accreditation Centers regarding the application of the Laboratory Combined MRA Mark. It allows for laboratories to apply this quality brand where applicable on internationally accepted test procedures and measurements. Laboratory test records are issued with the NCA logo and ILAC MRA marking. All these procedures are directed to achieve mutual cooperation with international organizations as a part of the World Trade Organization accession. The international acknowledgement of the national accreditation system will serve as the basis for bilateral arrangements between Kazakhstan and its trade partners, which will create favorable conditions for entering WTO.

